# Identification of SCAN Domain Zinc-Finger Gene ZNF449 as a Novel Factor of Chondrogenesis

**DOI:** 10.1371/journal.pone.0115169

**Published:** 2014-12-29

**Authors:** Keita Okada, Atsushi Fukai, Daisuke Mori, Yoko Hosaka, Fumiko Yano, Ung-il Chung, Hiroshi Kawaguchi, Sakae Tanaka, Toshiyuki Ikeda, Taku Saito

**Affiliations:** 1 Sensory & Motor System Medicine, Faculty of Medicine, The University of Tokyo, Tokyo, Japan; 2 Bone and Cartilage Regenerative Medicine, Faculty of Medicine, The University of Tokyo, Tokyo, Japan; 3 Center for Disease Biology and Integrative Medicine, Faculty of Medicine, The University of Tokyo, Tokyo, Japan; 4 Division of Transfusion Medicine, Faculty of Medicine, The University of Tokyo, Tokyo, Japan; 5 Sports Medicine and Orthopedics, Kanto Rosai Hospital, Japan Labour Health and Welfare Organization, Kanagawa, Japan; 6 Orthopedic Surgery, Tokyo Metropolitan Bokutoh Hospital, Tokyo, Japan; 7 Spine Center, Tokyo Shinjuku Medical Center, Japan Community Health care Organization, Tokyo, Japan; University of Saarland Medical School, Germany

## Abstract

Transcription factors SOX9, SOX5 and SOX6 are indispensable for generation and differentiation of chondrocytes. However, molecular mechanisms to induce the SOX genes are poorly understood. To address this issue, we previously determined the human embryonic enhancer of *SOX6* by 5′RACE analysis, and identified the 46-bp core enhancer region (CES6). We initially performed yeast one-hybrid assay for screening other chondrogenic factors using CES6 as bait, and identified a zinc finger protein ZNF449. ZNF449 and Zfp449, a counterpart in mouse, transactivated enhancers or promoters of *SOX6*, *SOX9* and *COL2A1*. *Zfp449* was expressed in mesenchyme-derived tissues including cartilage, calvaria, muscle and tendon, as well as in other tissues including brain, lung and kidney. In limb cartilage of mouse embryo, Zfp449 protein was abundantly located in periarticular chondrocytes, and decreased in accordance with the differentiation. Zfp449 protein was also detected in articular cartilage of an adult mouse. During chondrogenic differentiation of human mesenchymal stem cells, *ZNF449* was increased at an early stage, and its overexpression enhanced *SOX9* and *SOX6* only at the initial stage of the differentiation. We further generated *Zfp449* knockout mice to examine the *in vivo* roles; however, no obvious abnormality was observed in skeletal development or articular cartilage homeostasis. ZNF449 may regulate chondrogenic differentiation from mesenchymal progenitor cells, although the underlying mechanisms are still unknown.

## Introduction

In the initial step of skeletal development, undifferentiated mesenchymal cells are recruited into condensations and differentiate into chondrocytes that produce cartilage matrix proteins including type II collagen (*COL2A1*) and aggrecan [1]. This process is regulated by three members of the sex-determining region Y-type high mobility group box protein (SOX) family, a transcription factor SOX9, and its co-activators SOX5 and SOX6 [2]–[6]. The cartilage-specific deletion of *Sox9* results in a marked impairment of chondrogenesis in mouse embryos [7]. Although single null mice of *Sox5* or *Sox6* showed mild chondrodysplasia, double-null mice of Sox5 and Sox6 exhibit severe chondrodysplasia [6]. Molecular mechanisms to induce Sox9, Sox5 and Sox6 in immature mesenchyme are, however, poorly understood.

To address this issue, we previously determined the human embryonic enhancer of *SOX6* by 5′RACE analysis, and identified the 46-bp core enhancer region (CES6) [8]. We further revealed that CCAAT enhancer binding protein beta (C/EBPβ) transactivated CES6 [8]. In the present study, we first performed yeast one-hybrid assay for screening other chondrogenic factors using CES6 as bait, and identified a zinc finger protein ZNF449. ZNF449 was previously isolated from the human testis cDNA library and characterized as a nuclear protein which consists of 518 amino acids including an N-terminal SCAN domain and seven C2H2-type zinc finger motifs [9]; however, its roles have not been known at all. Here we report the expression pattern of Zfp449, a counterpart of ZNF449 in mice, and its function in chondrocyte differentiation of human mesenchymal stem cells (hMSCs). We further present an *in vivo* role of ZNF449 during skeletal development and articular cartilage homeostasis by deletion of *Zfp449* gene.

## Materials and Methods

### Yeast one-hybrid assay

We performed yeast one-hybrid assay using BD Matchmaker Library Construction & Screening Kits (Clontech) according to the manufacturer's protocol. Briefly, we cloned four tandem copies of CES6 into pHIS2 vector (pHIS2-CES6) as a reporter vector. We synthesized first-strand cDNA using total RNA from human trachea (Clontech), amplified double-strand (ds) cDNA by PCR with BD SMART III and CDS III anchors. pHIS2-CES6, ds cDNA, and pGADT7-Rec2 vector were co-transformed into yeast strain Y187, and positive clones were analyzed by PCR and DNA sequencing.

### Construction of expression vectors

We cloned ZNF449 and Zfp449 cDNA into pCMV-HA vector (Clontech), then constructed adenovirus vector expressing HA-tagged ZNF449 by the AdenoX Epression system (Clontech). All vectors were verified by DNA sequencing.

### Luciferase assay

We prepared the tandem-repeated CES6 reporter vector as previously described [8]. We amplified the *SOX9* promoter region (from −1,000 to 230 bp relative to the transcription start site), *COL2A1* promoter region (from −976 to 0 bp), *COL2A1* 3′ fragment containing enhancers (from +285 to 3,424 bp), *MMP13* (from −1,000 to 0 bp), and *ADAMTS5* (from −1,242 to +27 bp) by PCR using human genomic DNA as the template, and cloned them into pGL3-basic or pGL4.10[luc2] vector (Promega). Luciferase assays were performed in HeLa (Riken BRC) and SW1353 (American Type Culture Collection) cells as previously described [8].

### Chromatin immunoprecipitation (ChIP) assay

We performed the ChIP assay in SW1353 cells transfected with HA-tagged ZNF449 using OneDay ChIP Kit (Diagenode) according to the manufacturer's instructions. For immunoprecipitation, we used antibodies to HA (Abcam) and the normal rabbit IgG (Diagenode).

### Real-time RT-PCR

We collected organs and tissues from 8-week-old male C57BL/6 mice, homogenized them using Precellys24 tissue homogenizer (Bertin Technologies), and extracted total RNA from the lysates using RNeasy mini kits (Qiagen) according to the manufacturer's protocol. Real-time RT-PCR was performed as previously described [8].

### Histologial Analyses

Proximal tibia of mouse embryo (E18.5) and knee joint of 8-week-old mice were harvested, fixed with 4% paraformaldehyde for 24 hours, and 5-mm paraffin-embedded sections were prepared. H&E staining, Safranin-O staining, and immunofluorescence were performed as previously described [10], [11]. We used primary antibodies to ZNF449 (1∶100, Abcam), Sox9 (1∶100, Santa Cruz Biotechnology), Sox6 (1∶100, Santa Cruz Biotechnology), Col2a1 (1∶500, LSL) and GFP (1∶100, Abcam).

### Cell culture

hMSCs (Lonza) were maintained with the DMEM supplemented with 10% FBS, 100 U/mL penicillin and 100 µg/mL streptomycin. For chondrogenic differentiation, we formed a pellet from 2×10^5^ hMSCs and cultured in serum-free a-MEM (high glucose) supplemented with 6.25 µg/mL insulin, 6.25 µg/mL transferrin, 6.25 ng/mL selenite, 5.33 µg/mL linolate, 1.25 mg/mL bovine serum albumin, 10 ng/mL TGF-β1, 100 nM dexamethasone, and 50 µg/mL ascorbic acid–2-phosphate [12]. To introduce GFP or ZNF449, each adenoviral vector was transduced at 100 multiplicities of infection 2 days before the pellet formation. For primary cell cultures, we isolated chondrocytes from lower extremity epiphysis of *Zfp449* null and WT littermates (6 d) as previously described [13].

### Western Blotting

Cells were washed with ice-cold PBS, and proteins were extracted at 4°C with M-PER (Thermo Scientific). Equal amounts of protein were subjected to SDS-PAGE with 7.5–15% Tris-Glycin gradient gels onto PVDF membranes (Bio-Rad Laboratories, Inc.). After blocking with 5% skimmed milk/TBS-T, membranes were incubated with primary antibodies to HA (Abcam) or β-actin (Sigma-Aldrich), followed by HRP-conjugated goat anti–rabbit IgG (Promega). Immunoreactive bands were visualized with ECL (GE Healthcare) according to the manufacturer's instructions. The blots were stripped by incubating for 20 min in stripping buffer (2% SDS, 100 mM 2-mercaptoethanol, and 62.5 mM Tris-HCl, pH 6.7) at 50°C and reblotted with other antibodies.

### Generation of Zfp449 knockout (KO) mice

All experiments were performed according to a protocol approved by the Animal Care and Use Committee of the University of Tokyo (Permit Number: M-P12-131). All efforts were made to minimize suffering. We designed a targeting vector to replace exon3 and exon4 with EGFP and a neomycin resistance selection cassette. The targeting vector was linearized and introduced by electroporation into the embryonic stem (ES) cell derived from C57BL/6. Neomycin resistant colonies were isolated, and genomic DNA of the colonies was screened for homologous recombination by Southern blotting. Recombinant ES clones were injected into C57BL/6 mouse blastocysts to generate aggregation chimeras. Germline transmission was identified by PCR. Zfp449 KO mouse is deposited at the Center for Animal Resources and Development (CARD), Kumamoto University (http://card.medic.kumamoto-u.ac.jp/).

### Mouse osteoarthritis model

We performed the surgical procedure to create an experimental osteoarthritis model on 8-week-old male mice as previously described [11], [14]. Mice are operated under general anesthesia with isoflurane. Knees are exposed by a medial skin incision and the medial collateral ligament and medial meniscus are resected using a surgical microscope with close attention not to damage the articular cartilage. After irrigation with saline, the joint capsule and wound are closed with 6-0 nylon. 0.5% lidocaine was used for post-operative analgesia. 8 weeks after surgery, mice were euthanized by cervical dislocation and fixed with 4% paraformaldehyde. The knees were resected and embedded in paraffin for histological analyses.

### Statistical analysis

We performed statistical analyses of experimental data with the unpaired two-tailed Student *t* test.

## Results

### ZNF449 transactivates enhancers and/or promoters of chondrogenic factors

**Figure 1:**
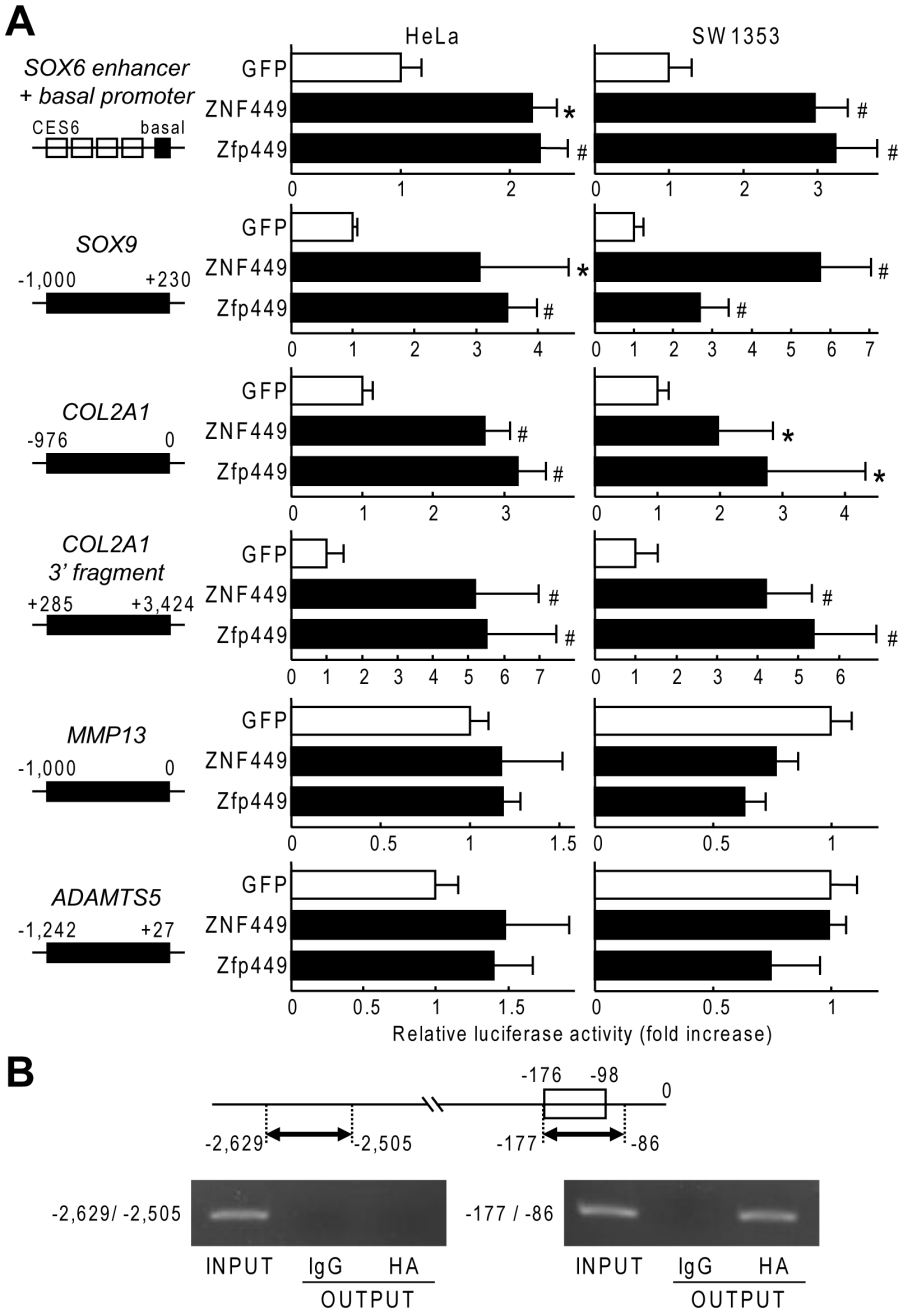
Transactivation of proximal promoters and/or enhancers of chondrocyte marker genes by ZNF449 (human) and Zfp449 (mouse). (A) Luciferase activities by the transfections of GFP, ZNF449 and Zfp449 into HeLa and SW1353 cells with a reporter construct containing the indicated fragment. Each luciferase activity is presented as fold increase relative to GFP control. Data are expressed as means (bars) ± SDs (error bars) for three wells/group. *P<0.05, ^#^ *P*<0.01 versus GFP. (B) ChIP assay with cell lysates of SW1353 cells transfected with HA-tagged ZNF449. PCR was performed with a primer set spanning the CES6 (−177 to −86 bp) or not spanning the CES6 (−2,629 to −2,505 bp) before (INPUT) and after immunoprecipitation with anti-HA (HA) or non-immune IgG (IgG).

For screening of transcription factors that bind to CES6, we initially performed a yeast one-hybrid assay using CES6 sequence as bait and a human trachea cDNA library as prey. In several positive clones, fragments of *ZNF449* coding sequence were identified. We confirmed that both human ZNF449 and Zfp449, a counterpart in mouse, significantly transactivated tandem-repeated CES6 by luciferase assay (Fig. 1A). ZNF449 and Zfp449 also transactivated proximal promoter regions of *SOX9* and *COL2A1*, and a 3′ fragment of *COL2A1* which contains potent enhancer regions (Fig. 1). Meanwhile, they did not alter the activity of *ADAMTS5* and *MMP13*, representative catabolic factors for cartilage (Fig. 1A). We then confirmed the *in vivo* binding of the ZNF449 protein to CES6 by ChIP assay (Fig. 1B).

### Expression pattern of Zfp449

**Figure 2:**
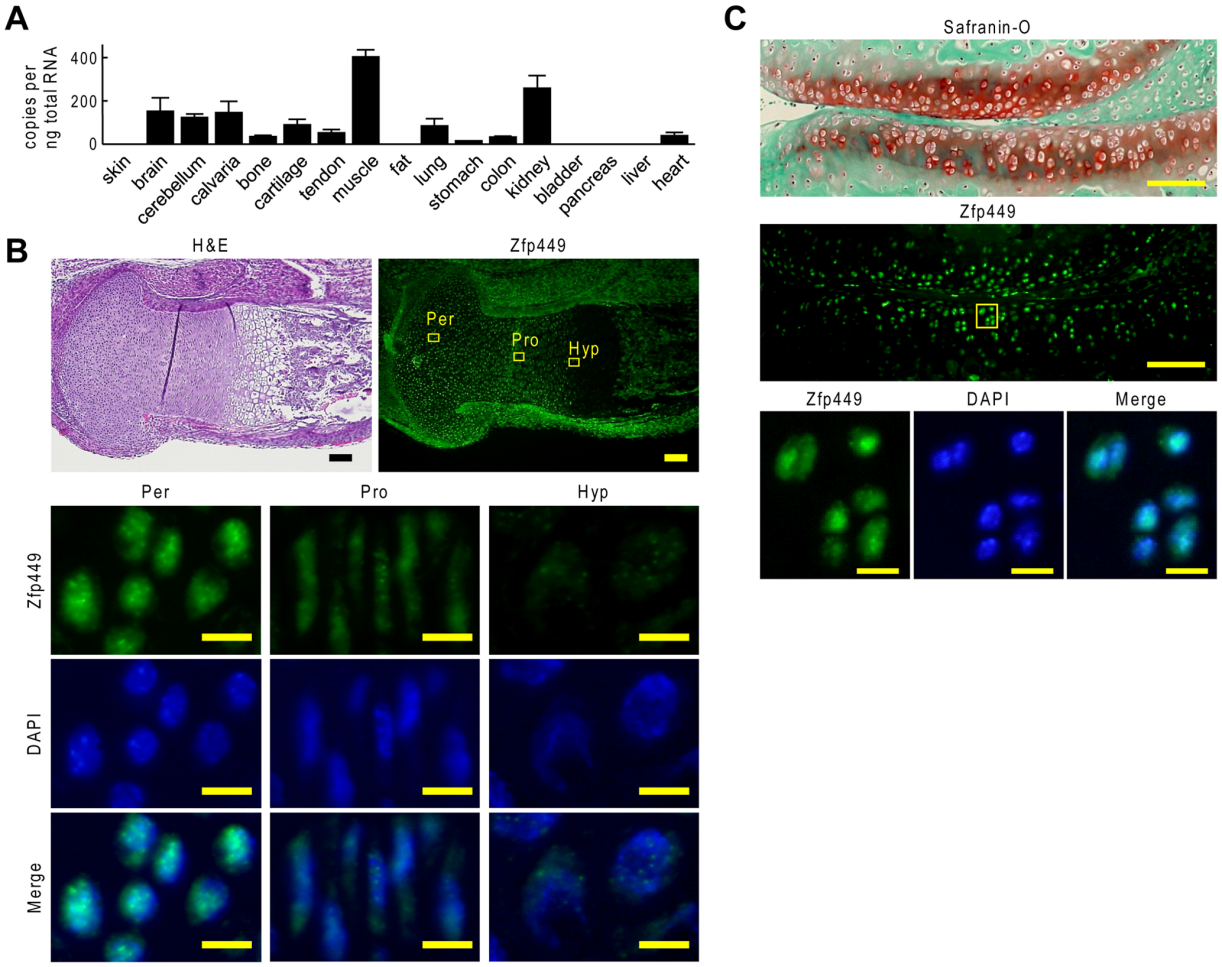
Expression pattern of Zfp449 in mouse tissues and cartilage. (A) mRNA level of *Zfp449* in mouse tissues. Data are expressed as means (bars) ± SDs (error bars) for three wells/group. (B) Immunofluorescence of Zfp449 in limb cartilage of mouse embryo. Inset boxes in the top panel indicate the regions of the bottom three rows representing periarticular (Per) zone, proliferative (Pro) zone, and hypertrophic (Hyp) zone. Scale bars, 100 µm (top), 20 µm (bottom). (C) Immunofluorescence of Zfp449 in articular cartilage of 8-week-old mouse knee joint. Inset box in middle panel indicates the regions of the bottom three rows. Scale bars, 100 µm (top and middle), 10 µm (bottom).

We then examined the expression pattern of *Zfp449* in adult mouse tissues by real-time RT-PCR. *Zfp449* was abundantly expressed in brain, cerebellum, calvaria, cartilage, muscle, lung, and kidney, but was scarcely expressed in skin, fat, bladder, pancreas, or liver (Fig. 2A).

In limb cartilage of a mouse embryo, Zfp449 protein was abundantly detected in the nuclei of periarticular chondrocytes and decreased in accordance with differentiation (Fig. 2B). In articular cartilage of an adult mouse, Zfp449 protein was widely detected in the nuclei of chondrocytes (Fig. 2C).

### ZNF449 regulates chondrocyte differentiation *in vitro*

**Figure 3:**
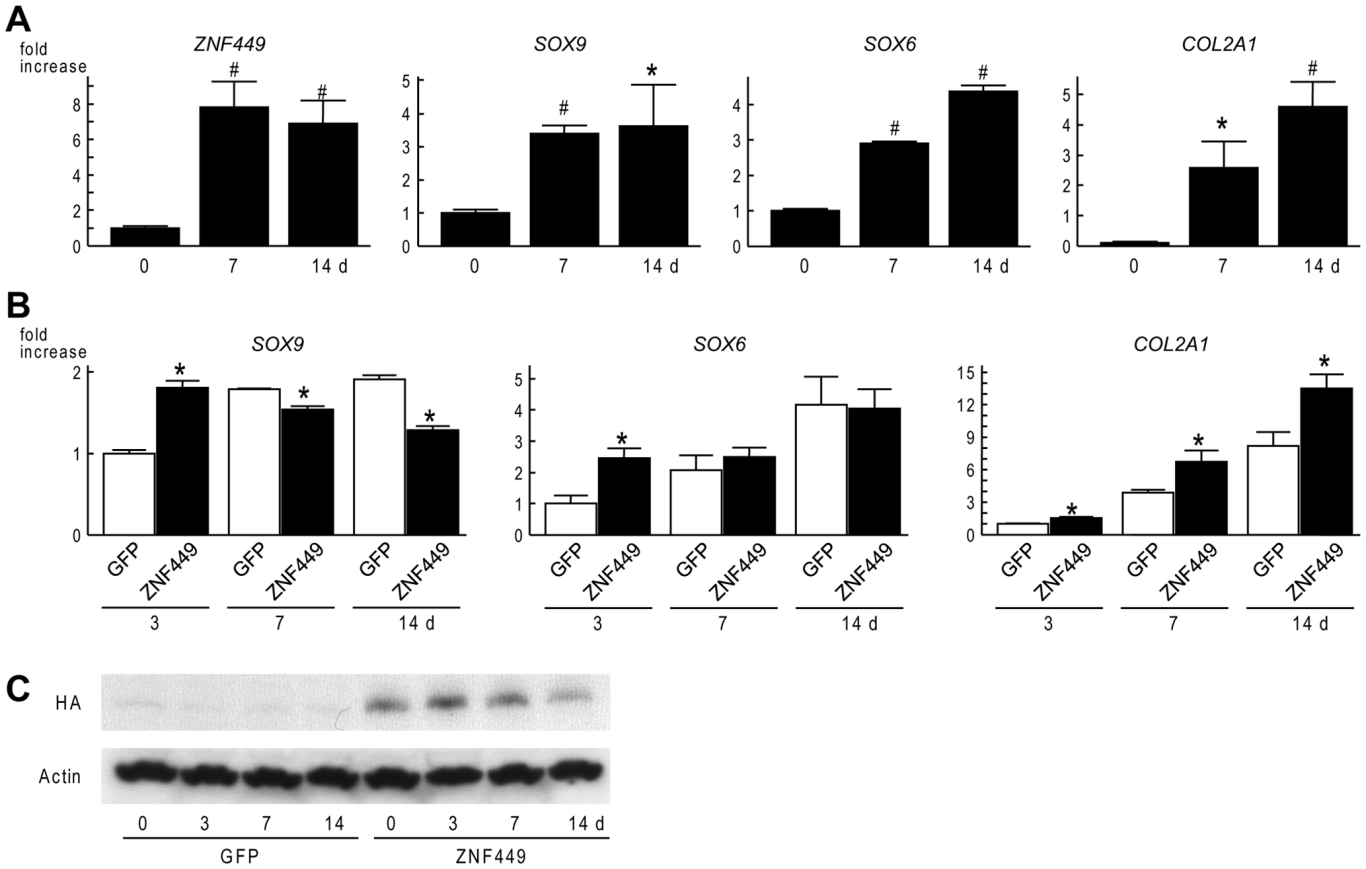
Enhancement of chondrogenic differentiation of hMSCs by ZNF449. (A) mRNA levels of *ZNF449, SOX9, SOX6 and COL2A1* during chondrogenic differentiation of hMSCs. Data are expressed as means (bars) ± SDs (error bars) for three wells/group. *P<0.05, ^#^ *P*<0.01 versus 0 d. (B) mRNA levels of *SOX9, SOX6 and COL2A1* during chondrogenic differentiation of hMSCs transduced with GFP or ZNF449 by adenovirus. Adenoviral transduction was performed 2 days before the differentiation. Data are expressed as means (bars) ± SDs (error bars) for three wells/group. *P<0.05 versus GFP. (C) Protein levels of the transduced ZNF449 (HA) and Actin during the differentiation of hMSC.

We next examined ZNF449 expression during chondrogenic differentiation of hMSCs. mRNA level of *ZNF449* was increased at day 7, simultaneously with that of SOX9, while those of *SOX6* and *COL2A1* were gradually increased during the differentiation (Fig. 3A). To examine roles of ZNF449 in chondrogenic differentiation, we introduced ZNF449 or GFP into hMSCs by adenovirus. When we started the differentiation 2 days after the adenoviral transduction, chondrogenic marker genes were significantly enhanced by ZNF449 overexpression at day 3 (Fig. 3B). mRNA level of *COL2A1* was enhanced throughout the differentiation; however, enhancement of *SOX6* was diminished after day 7, and *SOX9* was decreased in ZNF449 overexpressing cells after day 7 (Fig. 3B). Meanwhile, ZNF449 protein was overexpressed throughout the differentiation (Fig. 3C).

### Zfp449 knockout mice exhibit normal skeletal growth and articular cartilage homeostasis

**Figure 4:**
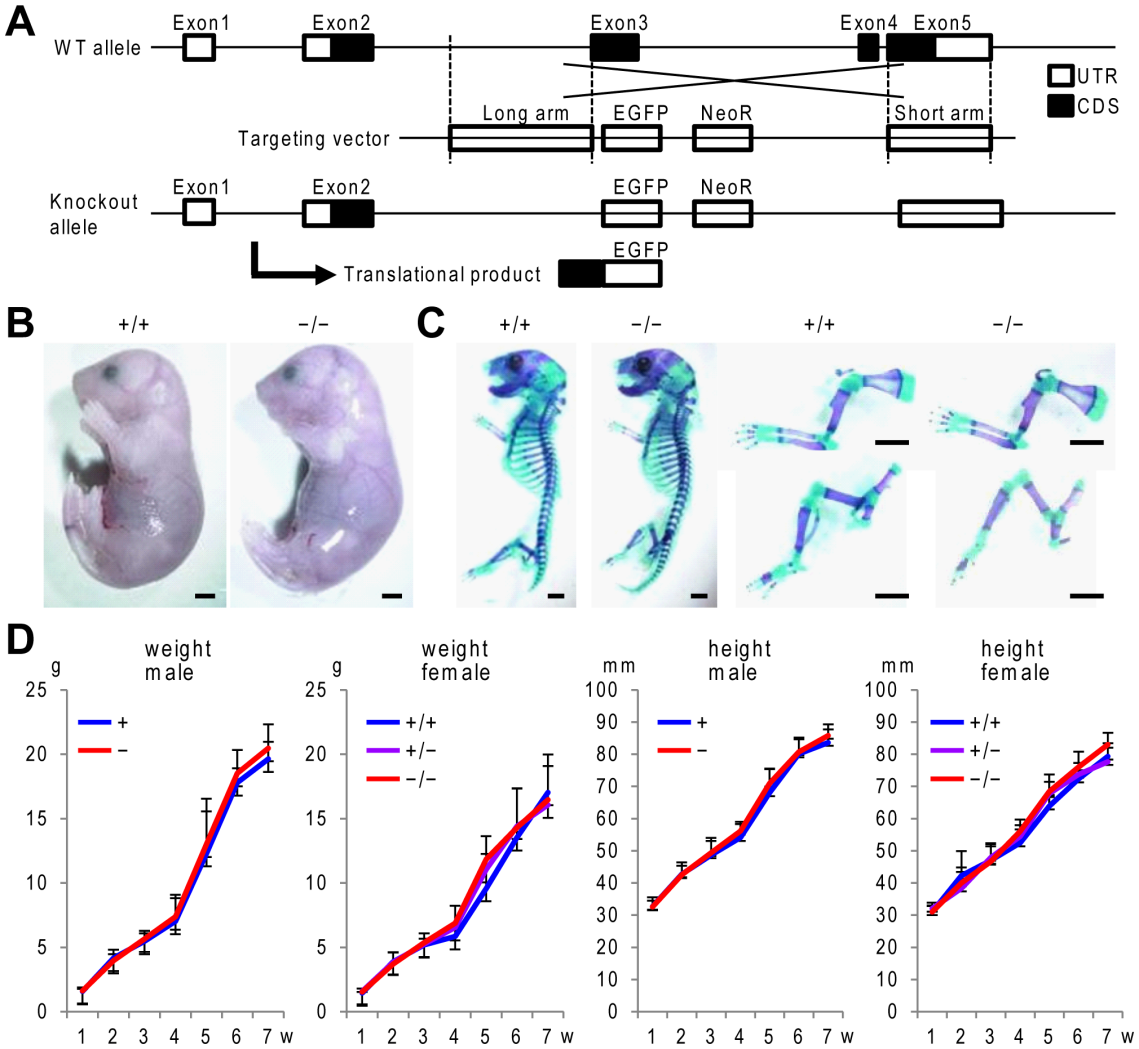
Generation of *Zfp449* knockout mice. (A) Schematic representation of the *Zfp449* locus and engineering of a *Zfp449* null allele by knocking in EGFP. UTR, untranslated region. CDS, coding sequence. NeoR, neomycin resistance gene. (B) Gross appearance of *Zfp449* null mice and WT littermate embryo (E18.5, female). Scale bars, 1 mm. (C) Double staining with Alizarin red and Alcian blue of the whole skeleton (left), upper extremities (right-top), and lower extremities (right-bottom) of *Zfp449* null mice and WT littermate embryo (E18.5, female). Scale bars, 1 mm. (D) Growth curves of *Zfp449* knockout littermates (+ and − for male, and +/+, +/−, and −/− for female). Height indicates nose-anus length. *P<0.05 versus GFP.

To further know *in vivo* functions of Zfp449, we generated knockout mice by recombination of exon 3 and 4 with GFP and a neomycin resistance gene (Fig. 4A). *Zfp449* null male and female mice were born without obvious abnormality, and exhibited normal skeletal growth (Fig. 4B-D). They were fertile, and showed normal behavior throughout their lifespan up to 17 months.

**Figure 5:**
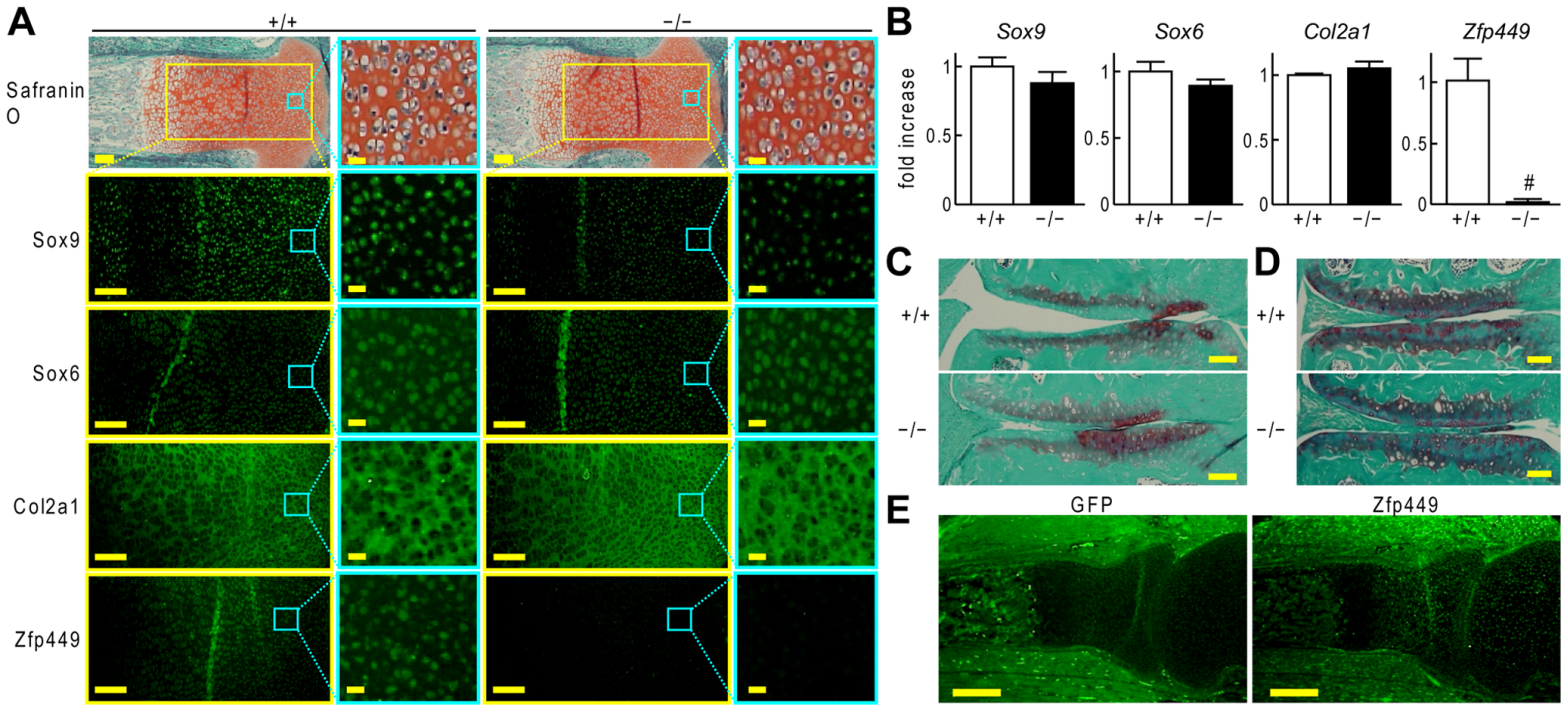
Histological analyses of *Zfp449* knockout mice. (A) Safranin-O staining and immunofluorescence of Sox9, Sox6, Col2a1 and Zfp449 in proximal tibia of *Zfp449* null (−/−) and WT littermate embryos (+/+) (E18.5). Enlarged immunofluorescence figures on the left are from the areas indicated in the yellow inset box on Safranin-O staining. The further magnified views on the right are from the areas indicated in the blue inset boxes. Scale bars, 100 µm (Safranin-O stainings and immunofluorescence images), 10 µm (maginified views of immunofluorescence). (B) mRNA levels of *Sox9*, *Sox6*, *Col2a1* and *Zfp449* in primary chondrocytes from *Zfp449* null and WT littermate mice (6 d). Data are expressed as means (bars) ± SDs (error bars) for three wells/group. ^#^ *P*<0.01 versus WT. (C) Safranin-O staining of knee joints in *Zfp449* null mice and WT littermates 8 weeks after the surgical induction of OA. Scale bars, 100 µm. (D) Safranin-O staining of knee joints in 17-month-old *Zfp449* null mice and WT littermates. Scale bars, 100 µm. (E) Immunofluorescence of GFP and Zfp449 in elbow joints of *Zfp449* heterozygous mutant. Scale bars, 200 µm.

Next we performed histological analyses of *Zfp449* null limb cartilage. Although Sox9 was decreased and Zfp449 was efficiently suppressed in *Zfp449* null mice, Safranin-O staining and other anabolic marker genes were not altered (Fig. 5A). In primary chondrocytes, there were no significant differences in mRNA expression of anabolic maker genes (Fig. 5B). When we created a surgical osteoarthritis model [14], no significant difference was observed in osteoarthritis development (Fig. 5C). We then went on to look for any age-related changes in articular cartilage, but also found no significant difference in the two genotypes up to 16 months of age (Fig. 5D). Finally, we confirmed that expression pattern of GFP was similar to that of Zfp449 in a heterozygous mutant (Fig. 5E), indicating that the heterozygous mutant is useful for Zfp449 reporter mice.

## Discussion

In the present study, we identified ZNF449 as a candidate of CES6 activator. The protein consists of 518 amino acids including an N-terminal SCAN domain and seven C2H2-type zinc finger motifs [9]. ZNF449 protein is located in the nucleus, and is predicted to function as a transcription factor [9]. The present data also showed that Zfp449 was primarily located in the nuclei of periarticular chondrocytes of mouse embryonic limb, and of adult articular cartilage (Fig. 2B,C). The *in vitro* gain-of-function study showed its potent function in promoting chondrogenic differentiation of hMSCs (Fig. 3B). However, *Zfp449* null mice displayed normal skeletal development and articular cartilage homeostasis, in contrast to the results obtained by the *in vitro* experiments. Since there are numerous proteins which contain zinc finger motifs, the normal phenotype of *Zfp449* null mice may be due to compensation by other factors which have similar domains or motifs to Zfp449.

Recently, zinc fingers have been recognized to have a protein-binding ability, as well as a DNA-binding ability [15]. Structural studies of ZNF complexes revealed diversity of partner proteins [15]. In the present study, ZNF449 was expressed in various mesenchymal and mesenchyme-derived cells including hMSCs, bone, cartilage, tendon and muscle (Figs. 2A, 3A). Considering these data, ZNF449 may regulate differentiation of the mesenchymal cells with different partners, and need some specific co-factors to exert the chondrogenic function.

The *Zfp449* null mice did not show obvious abnormalities during skeletal development; nevertheless, expression of Sox9 protein was decreased in embryonic limb cartilage of *Zfp449* null mice (Fig. 5A). Considering that *Sox9* mRNA level was not significantly decreased in chondrocytes from 6-day-old Zfp449 null mice (Fig. 5B) and that enhancement of SOX9 expression by ZNF449 was observed only in the early stage of chondrogenic differentiation of hMSC (Fig. 3B), Zfp449 may be required for SOX9 expression in only a limited period during the early differentiation stage.

In the present study, ZNF449 enhanced *COL2A1* expression throughout the chondrogenic differentiation of hMSCs; meanwhile, enhancement of *SOX6* was diminished at day 7, and *SOX9* expression was decreased after day 7, in contrast to that at day 3 (Fig. 3B). These discrepancies imply that ZNF449 is involved with induction of the three genes by different mechanisms: i.e., by forming different protein complexes with different co-factors. Considering that ZNF449 contributes to enhanced expression of *SOX9* and *SOX6* only at day 3, the co-factors of ZNF449 which are involved with the transcriptional induction of the SOX genes may be expressed or function only in the early stage of chondrogenic differentiation. In contrast, enhancement of *COL2A1* expression by ZNF449 might be independent of SOX9 or SOX6, particularly in the later stage. Elucidation of these protein complexes with ZNF449 may lead us to know the molecular mechanism of SOX9 induction, which is yet to be understood.
